# Lactopeptine

**Published:** 1878-01-20

**Authors:** 


Lactopeptine. —- Among the recent efforts of pharmacy is that of the imitation of nature's processes in substituting the gastric and pancreatic juices where deficient. For this purpose we have Ingluvin, Pepsin, Lactopeptine, etc. In the article on dyspepsia, in this number, cases are given of success in the use of Lactopeptine.

